# Liquid Biopsy in Gastrointestinal Cancers: How Close Are We to Reaching the Clinic?

**DOI:** 10.3390/cancers15102831

**Published:** 2023-05-19

**Authors:** Julie Earl, Agapi Kataki, Bozena Smolkova

**Affiliations:** 1 Molecular Epidemiology and Predictive Tumor Markers Group, Ramón y Cajal Health Research Institute (IRYCIS), Carretera Colmenar Km 9100, CIBERONC, 28034 Madrid, Spain; 2 1st Department of Propaedeutic Surgery, National and Kapodistrian University of Athens, Vasilissis Sofias 114, 11527 Athens, Greece; akataki@med.uoa.gr; 3 Department of Molecular Oncology, Cancer Research Institute, Biomedical Research Center of the Slovak Academy of Sciences, Dubravska Cesta 9, 845-05 Bratislava, Slovakia; bozena.smolkova@savba.sk

Gastrointestinal (GI) cancers are malignancies that develop within the digestive system and account for one in four cancer cases according to WHO data [1]. Esophageal, gastric, liver, pancreatic, colon and rectum cancers represent the most common types of GI malignancies, whereas small bowel cancer, gallbladder cancer, and carcinoid tumors are less common. They present a geographical and temporal heterogeneity and risk factors that involve both genetic variation and environmental exposure. Despite advances in treatment which often involves surgery, chemotherapy, radiation, and, more recently, immunotherapy in certain patients, they are major contributors to neoplasm-related deaths, accounting for one in three cancer related deaths globally [2]. Both treatment strategy and prognosis depend on several factors, including the type and stage of cancer and each patient’s overall health and medical history. In all cases, the assessment of response to treatment or progression of the disease is mainly conducted using the Response Evaluation Criteria in Solid Tumors (RECIST v1.1), where the application of imaging techniques is essential, as the current sophisticated imaging technology provide excellent diagnostic accuracy [3,4]. In addition, soluble blood markers such as CA19-9 and CEA combined with complete blood counts and biochemical blood tests are also routinely used to determine disease stage and response to treatment. However, as cancer mortality is mainly due to metastatic spread, the analysis of factors related to its metastatic potential represent a useful source of information, and thus, liquid biopsy has emerged as a non-invasive tool that can complement traditional approaches. The term “liquid biopsy” was first introduced by Klaus Pantel and Catherine Alix-Panabières in 2010 [5] and is defined as the detection and analysis of molecules (e.g., protein, DNA, and RNA), cells, or extracellular vesicles (EVs, e.g., exosomes) that originate from the primary tumor in blood or in other biological fluids [6]. Through liquid biopsy, dynamic changes in tumor cells can be screened in real-time, with sequential monitoring of disease, determination of the metastatic relapse risk, and stratification of patients being some of its numerous potential applications in clinical practice, promoting precision medicine in patients with GI malignancies [5].

The application of liquid biopsy in these types of cancers and more specifically in colorectal cancer (CRC) and gastro-esophageal cancer is the focus of this Special Issue. Four original articles on the use of EVs and cell-free DNA (cfDNA)—including diagnostic plasma variants as predictive markers of these tumors and one review on the use of circulating tumor DNA (ctDNA) in the assessment of minimal residual disease (MRD) in CRC—are included. The technical aspects and challenges behind the clinical application of liquid biopsy are especially underlined. The detection of disseminated and residual disease before and after surgical resection, respectively, are important clinical issues faced by surgeons and medical oncologists treating digestive tumors. The term MRD is used to describe the very few cancer cells which remain in the patient’s body during or after cancer treatment, which are below detectable levels after oncological treatment using routine laboratory methods [7]. MRD was first recognized and reported in the 1950s, with a number of related articles on various tumor types, with an explosion of articles at the beginning of the 21st century, particularly in lymphoma and leukemia. Over 200,000 articles, including over 400 reviews, have been published on MRD since the turn of the century, with 258 original articles and 34 review articles focused on GI cancer, according to PUBMED (last access 26 April 2023).

The study of Wallander et al. [8] describes the use of panel sequencing of DNA derived from both tumor tissue and ctDNA recovered before and after surgery, from patients diagnosed with gastro-esophageal cancer. Importantly, both tumor-informed and tumor-agnostic approaches are applied to filter variants, with the sequencing of matched white blood cells from selected cases also included in this study, allowing the exclusion of clonal hematopoietic variants. In 55% of cases, tissue-verified cancer-associated variants were detected in ctDNA derived from liquid biopsy via a tumor-agnostic approach; the percentage of somatic variants detected increased to 59% of cases, with unique molecular identifiers used to differentiate the true from technical artefacts. In their cohort, the detection of tumor associated variants correlated with shorter overall survival and shorter time to disease progression. Furthermore, their study points out the advantage of digital droplet PCR as a very sensitive method for the detection of somatic variants in cfDNA derived from liquid biopsy.

On a more technical note, Kalmár et al. [9], describe a study of whole-exome sequencing (WES) of matched tumor tissue and cfDNA from colorectal adenoma and CRC patients, with targeted panel sequencing of cfDNA in a subgroup of patients. This study shows a high correlation between matched tissue and plasma variant allele frequencies based on WES results, with the somatic mutation landscape of benign and malignant colon lesions showing distinct patterns. Furthermore, their claim is that a high coverage panel sequencing of targeted regions can be more efficient, as it could reveal over half of the tumor somatic variants falling on its targeted regions, whereas only 20% were recovered by WES. Similarly to Wallander et al., this study also underlines the value of digital droplet PCR as a very sensitive method for the detection of somatic variants in liquid biopsy derived material.

In CRC patients, metastasis prediction is also a major issue as many patients progress after treatment for localized disease [10]. The study by Brocco et al. [11], emphasizes the prognostic and predictive value of EVs presence in blood and especially those expressing CD133 in metastatic CRC patients. Increased blood concentrations of CD133+ EVs at baseline correlated with reduced overall survival and overall response rate to first-line systemic therapy [11]. 

Patient stratification via the detection of MRD after a curative-intent resection has been a challenge in CRC over the last years [12]. In this Special Issue, Chakrabarti et al. [13] provide a comprehensive and informative review of MRD assessment via cfDNA in the liquid biopsy, which is crucial to avoid under and over treatment with post-surgical adjuvant therapy in the clinic. There are many ongoing ctDNA-guided adjuvant clinical trials that will likely result in the introduction of this advanced technology in the post-surgical treatment strategies of CRC patients in the near future.

The research study of Hofste et al. is focused on patients with locally advanced esophageal cancer and represents a well-designed and executed study for the use of ctDNA detection to predict residual disease during treatment [14]. Esophageal cancer is the seventh most common cancer and the sixth most common cause of cancer-related mortality [15]. In locally advanced esophageal cancer, neoadjuvant chemoradiotherapy followed by surgery is often the treatment of choice [15]. However, recurrence after neoadjuvant therapy and the prediction of distant metastasis are major challenges in this disease. Somatic mutations detected in tumor tissue after neoadjuvant chemoradiotherapy and surgery were used to measure the presence of ctDNA in serially collected plasma samples at diagnosis, prior to chemoradiotherapy treatment and surgery by ultradeep sequencing. The detection of ctDNA before chemoradiotherapy treatment in 56% of cases was associated with tumor stage and volume, whereas ctDNA after chemoradiotherapy treatment and before surgery was detected in 10% of patients and was independently associated with disease recurrence. These results indicate that ctDNA detection before surgery in patients with locally advanced disease could represent a stratification tool in the assessment of recurrence risk after neoadjuvant chemoradiotherapy and surgery in patients with esophageal cancer.

Liquid biopsy has emerged in clinical oncology as an important tool for patient management, and is on the verge of being implemented into the clinical practice [16], as the studies presented in this Special Issue demonstrate, with cfDNA detection being the most likely marker to win the race, at least in the case of CRC. Still, there are issues that need to be resolved, including a final consensus regarding the technique of choice for variant detection in cfDNA, the optimal pre-analytical procedures and validated SOPs [17], an efficient and effective sample traceability and an accredited quality management system. Recently, the European Society of Medical Oncology (ESMO) has published guidelines including the use of ctDNA for the detection of somatic mutations to identify actionable therapeutic targets [18]. Even so, the jury appears to still be out regarding the use of plasma or serum for ctDNA detection, although plasma seems to be the best choice, as the potential to detect variants with a low allele frequency in serum are limited [19]. The clinical significance of clonal hematopoiesis (CH) variants detected in whole blood should also be taken under consideration when applying this technology in the clinic. CH-related mutations were reported to be present in over 35% of healthy individuals and cancer cases [20] and found to be more frequent in older patients.

The prognosis of CRC has improved significantly in recent years due to liquid biopsy-based early detection strategies, such as the fecal occult blood test or fecal immunochemical test [21,22]. However, the expectations for the implementation of liquid biopsy based biomarkers in the clinical setting of GI malignancies are far from being fulfilled and therefore our work must go on.

## References

[1] de Martel C., Georges D., Bray F., Ferlay J., Clifford G.M. (2020). Global Burden of Cancer Attributable to Infections in 2018: A Worldwide Incidence Analysis. Lancet Glob. Health.

[2] Arnold M., Abnet C.C., Neale R.E., Vignat J., Giovannucci E.L., McGlynn K.A., Bray F. (2020). Global Burden of 5 Major Types of Gastrointestinal Cancer. Gastroenterology.

[3] Eisenhauer E.A., Therasse P., Bogaerts J., Schwartz L.H., Sargent D., Ford R., Dancey J., Arbuck S., Gwyther S., Mooney M. (2009). New Response Evaluation Criteria in Solid Tumours: Revised RECIST Guideline (Version 1.1). Eur. J. Cancer.

[4] Schäfer V.S., Jin L., Schmidt W.A. (2020). Imaging for Diagnosis, Monitoring, and Outcome Prediction of Large Vessel Vasculitides. Curr. Rheumatol. Rep..

[5] Alix-Panabières C., Pantel K. (2016). Clinical Applications of Circulating Tumor Cells and Circulating Tumor DNA as Liquid Biopsy. Cancer Discov..

[6] Pantel K., Alix-Panabières C. (2010). Circulating Tumour Cells in Cancer Patients: Challenges and Perspectives. Trends Mol. Med..

[7] Luskin M.R., Murakami M.A., Manalis S.R., Weinstock D.M. (2018). Targeting Minimal Residual Disease: A Path to Cure?. Nat. Rev. Cancer.

[8] Wallander K., Haider Z., Jeggari A., Foroughi-Asl H., Gellerbring A., Lyander A., Chozhan A., Cuba Gyllensten O., Hägglund M., Wirta V. (2023). Sensitive Detection of Cell-Free Tumour DNA Using Optimised Targeted Sequencing Can Predict Prognosis in Gastro-Oesophageal Cancer. Cancers.

[9] Kalmár A., Galamb O., Szabó G., Pipek O., Medgyes-Horváth A., Barták B.K., Nagy Z.B., Szigeti K.A., Zsigrai S., Csabai I. (2023). Patterns of Somatic Variants in Colorectal Adenoma and Carcinoma Tissue and Matched Plasma Samples from the Hungarian Oncogenome Program. Cancers.

[10] Argilés G., Tabernero J., Labianca R., Hochhauser D., Salazar R., Iveson T., Laurent-Puig P., Quirke P., Yoshino T., Taieb J. (2020). Localised Colon Cancer: ESMO Clinical Practice Guidelines for Diagnosis, Treatment and Follow-Up. Ann. Oncol..

[11] Brocco D., Simeone P., Buca D., Di Marino P., De Tursi M., Grassadonia A., De Lellis L., Martino M.T., Veschi S., Iezzi M. (2022). Blood Circulating CD133+ Extracellular Vesicles Predict Clinical Outcomes in Patients with Metastatic Colorectal Cancer. Cancers.

[12] Badia-Ramentol J., Linares J., Gómez-Llonin A., Calon A. (2021). Minimal Residual Disease, Metastasis and Immunity. Biomolecules.

[13] Chakrabarti S., Kasi A.K., Parikh A.R., Mahipal A. (2022). Finding Waldo: The Evolving Paradigm of Circulating Tumor DNA (CtDNA)—Guided Minimal Residual Disease (MRD) Assessment in Colorectal Cancer (CRC). Cancers.

[14] Hofste L.S.M., Geerlings M.J., von Rhein D., Tolmeijer S.H., Weiss M.M., Gilissen C., Hofste T., Garms L.M., Janssen M.J.R., Rütten H. (2022). Circulating Tumor DNA-Based Disease Monitoring of Patients with Locally Advanced Esophageal Cancer. Cancers.

[15] Obermannová R., Alsina M., Cervantes A., Leong T., Lordick F., Nilsson M., van Grieken N.C.T., Vogel A., Smyth E.C. (2022). Oesophageal Cancer: ESMO Clinical Practice Guideline for Diagnosis, Treatment and Follow-Up. Ann. Oncol..

[16] De Mattos-Arruda L., Siravegna G. (2021). How to Use Liquid Biopsies to Treat Patients with Cancer. ESMO Open.

[17] Earl J., Calabuig-Fariñas S., Sarasquete M.E., Romay L.M., Lopez-Tarruella S., Paricio B.B., Rodríguez M., Leoz K.V., Porto M.D., Tarazona N. (2022). A Standardized Liquid Biopsy Preanalytical Protocol for Downstream Circulating-Free DNA Applications. J. Vis. Exp..

[18] Heitzer E., van den Broek D., Denis M.G., Hofman P., Hubank M., Mouliere F., Paz-Ares L., Schuuring E., Sültmann H., Vainer G. (2022). Recommendations for a Practical Implementation of Circulating Tumor DNA Mutation Testing in Metastatic Non-Small-Cell Lung Cancer. ESMO Open.

[19] Pittella-Silva F., Chin Y.M., Chan H.T., Nagayama S., Miyauchi E., Low S.K., Nakamura Y. (2020). Plasma or Serum: Which Is Preferable for Mutation Detection in Liquid Biopsy?. Clin. Chem..

[20] Chang C.M., Lin K.C., Hsiao N.E., Hong W.A., Lin C.Y., Liu T.C., Chang Y.S., Chang J.G. (2022). Clinical Application of Liquid Biopsy in Cancer Patients. BMC Cancer.

[21] Robertson D.J., Lee J.K., Boland C.R., Dominitz J.A., Giardiello F.M., Johnson D.A., Kaltenbach T., Lieberman D., Levin T.R., Rex D.K. (2017). Recommendations on Fecal Immunochemical Testing to Screen for Colorectal Neoplasia: A Consensus Statement by the US Multi-Society Task Force on Colorectal Cancer. Gastroenterology.

[22] Mousavinezhad M., Majdzadeh R., Sari A.A., Delavari A., Mohtasham F. (2016). The Effectiveness of FOBT vs. FIT: A Meta-Analysis on Colorectal Cancer Screening Test. Med. J. Islam. Repub. Iran.

